# Potential Therapeutic Approach of Melatonin against Omicron and Some Other Variants of SARS-CoV-2

**DOI:** 10.3390/molecules27206934

**Published:** 2022-10-16

**Authors:** Rahima Begum, A. N. M. Mamun-Or-Rashid, Tanzima Tarannum Lucy, Md. Kamruzzaman Pramanik, Bijon Kumar Sil, Nobendu Mukerjee, Priti Tagde, Masayuki Yagi, Yoshikazu Yonei

**Affiliations:** 1 Department of Microbiology, Gono Bishwabidyalay, Dhaka 1344, Bangladesh; 2 Anti-Aging Medical Research Center, Graduate School of Life and Medical Sciences, Doshisha University 1-3 TataraMiyakodani, Kyoto 610-0394, Japan; 3 Glycative Stress Research Center, Graduate School of Life and Medical Sciences, Doshisha University 1-3 Tatara Miyakodani, Kyoto 610-0394, Japan; 4 Department of Environmental & Occupational Health, School of Public Health, University of Pittsburgh, 130 De Soto Str., Pittsburgh, PA 15231, USA; 5 Microbiology and Industrial Irradiation Division, Institute of Food and Radiation Biology, Atomic Energy Research Establishment, Savar 1349, Bangladesh; 6 Department of Microbiology, Ramakrishna Mission Vivekananda Centenary College, Kolkata 700118, India; 7 Department of Health Sciences, Novel Global Community Educational Foundation, Sydney 37729, Australia; 8 Patel College of Pharmacy, Madhyanchal Professional University, Bhopal 462044, India

**Keywords:** Omicron variant (B.529), COVID-19 disease, SARS-CoV-2, oxidative stress, melatonin, antioxidant, therapeutic approach

## Abstract

The Omicron variant (B.529) of COVID-19 caused disease outbreaks worldwide because of its contagious and diverse mutations. To reduce these outbreaks, therapeutic drugs and adjuvant vaccines have been applied for the treatment of the disease. However, these drugs have not shown high efficacy in reducing COVID-19 severity, and even antiviral drugs have not shown to be effective. Researchers thus continue to search for an effective adjuvant therapy with a combination of drugs or vaccines to treat COVID-19 disease. We were motivated to consider melatonin as a defensive agent against SARS-CoV-2 because of its various unique properties. Over 200 scientific publications have shown the significant effects of melatonin in treating diseases, with strong antioxidant, anti-inflammatory, and immunomodulatory effects. Melatonin has a high safety profile, but it needs further clinical trials and experiments for use as a therapeutic agent against the Omicron variant of COVID-19. It might immediately be able to prevent the development of severe symptoms caused by the coronavirus and can reduce the severity of the infection by improving immunity.

## 1. Introduction

COVID-19 is a disease caused by a recently discovered coronavirus called pathogenic SARS-CoV-2. It is contagious and quickly spreads from person to person [1]. The World Health Organization (WHO) states that the global COVID-19 pandemic began in March 2020. Compared to other variants of the disease, Omicron SARS-CoV-2 (B.529) exhibits a larger number of mutations [2]. In the Omicron variant, mutations have been found fifteen times [3]. COVID-19 has not yet been given a specific pharmacological treatment. A variety of pharmacological antiviral drugs and other therapeutic options have been clinically investigated for the treatment of COVID-19 disease, such as convalescent plasma therapy, monoclonal antibodies, and some immunomodulatory drugs. TheCOVID-19 disease scenario developed rapidly, and researchers tried to quickly identify an effective agent. Although these therapeutic approaches primarily succeeded in reducing COVID-19 disease progression, some of the agents are not functionally active for the treatment of the disease. Several clinical trials and investigations were forced to avoid using these drugs and agents as a therapeutic approach. In addition, a lack of safety, especially for children and pregnant women, along with long-term action, unknown efficacy and tolerance, and significant side effects were observed. Due to these complications, the FDArestricted thesetherapeutic agents [4,5,6]. Thus, COVID-19 still lacks a particular treatment. The most important conditions of SARS-CoV-2 infection are excessive inflammatory responses and cytokine storm [7]. It has been established that elevated levels of the pro-inflammatory cytokines IL-1, IL-6, and TNF-αare what primarily induce COVID-19 infection symptoms [8]. In most cases, the human body responds to infection and inflammation at the same time. To combat this situation, a broader and less virus-specific therapy that focuses on the severe symptoms of viral infection should be considered. Currently, researchers strongly advocate for melatonin’s therapeutic potential and application in the treatment of COVID-19 [9].

**Figure 1:**
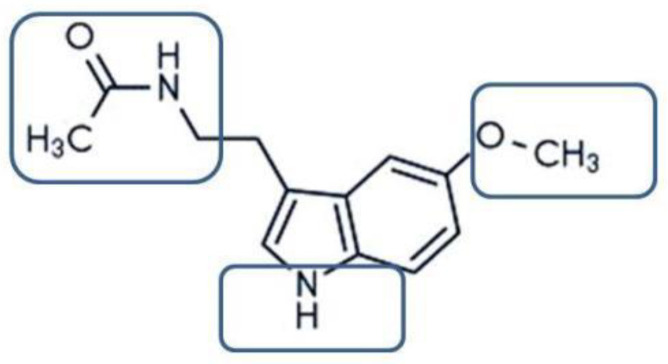
Functional group of melatonin, both hydrophilic and lipophilic innature, whichallows itto exhibit strong antioxidant activity.

Melatonin (*N*-acetyl-5-methoxytryptamine) is often referred to as an endogenous neurohormone and is primarily biosynthesized from tryptophan in the pineal gland in response to darkness and discharged into the cerebrospinal fluid, blood, and almost all the organsand tissuesof the body [10,11]. Its production and presence are also in the retina, bone marrow, the gastrointestinal tract, and several other organs [12,13,14,15,16,17,18,19]. Melatonin plays positive roles in physiological processes, including the sleep cycle, mood, anxiety, appetite, immune response, and cardiac functions [20]. It has been used to treat sleep disorders, atherosclerosis, respiratory diseases, and viral infections [11]. Although it does not have direct virucidal effects on COVID-19 and its Omicron (B.529) variant, it demonstrates anti-viral actions based on its anti-inflammation, anti-oxidation, and immune-enhancing properties [21]. It is clearly documented from the chemical point of view that melatonin is highly specified as an antioxidative agent. The functional chemical scaffold with a three-amide group and a five-alkoxy group is primarily responsible for the amphiphilicity of this molecule (Figure 1). Based on thechemical structure, several studies proved that melatonin could penetrate the biological membrane and enter any cellular and subcellular compartment, as well as offer protection against oxidative stress in various cell compartments [22,23].

**Figure 2:**
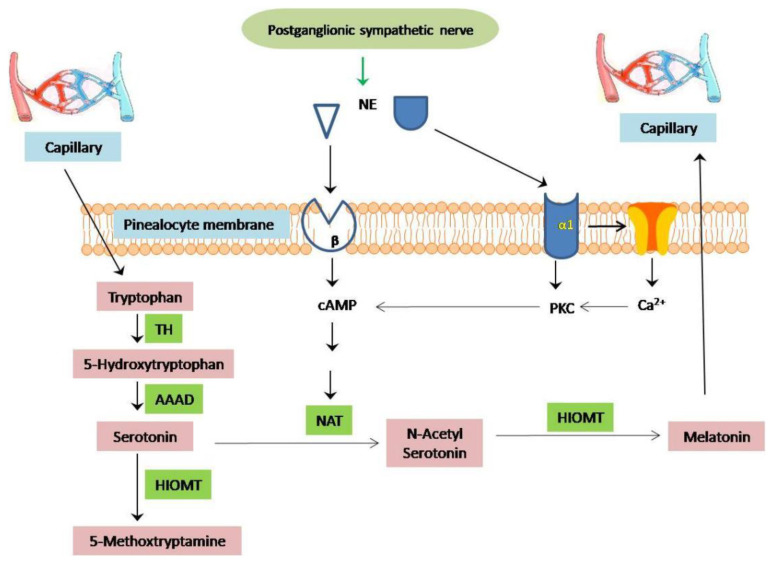
Biosynthesis process of melatonin from tryptophan. TH, tryptophan-5-hydroxylase; AAAD, aromatic amino acid decarboxylase; NAT, arylalkylamine*N*-acetyltransferase; HIOMT, hydroxyindole-O-methyltransferase.

In general, the synthesis, release, and distribution of melatonin are maintained under the sympathetic innervation with pinealocytes (the major cells in the pineal gland) [24]. This whole controlling system is dependent on the photoperiod and four enzymes, namelytryptophan hydroxylase (TH), aromatic acid decarboxylase (AAAD), N-acetyltransferase (NAT), and hydroxy indole-O-methyltransferase (HIOMT), which are responsible for converting tryptophan to melatonin (*N*-acetyl-5-methoxy-tryptamine). In darkness, these nerve endings release norepinephrine (NE), which acts primarily on ß-adrenergic receptors (ß) and alpha 1-adrenergic receptors (α1) to promote the nocturnal synthesis of melatonin. The synthesis of melatonin from tryptophan is a multi-step process. Tryptophan is hydroxylated by tryptophan-5-hydroxylase (TH) to form 5-hydroxytryptophan, which is subsequently decarboxylated to 5-hydroxytryptamine (serotonin) by aromatic amino acid decarboxylase (AAAD). Serotonin is N-acetylated by N-acetyltransferase (NAT) to form N-acetylserotonin, which is converted to N-acetyl-5-methoxytryptamine (melatonin) by N-acetylserotonin-O-methyltransferase (ASMT, also called hydroxy indole-O-methyltransferase or HIOMT). Norepinephrine induces its α1/β-adrenoceptors, whichactivate the adenylate cyclase–cAMP system. Thus, intracellular levels of the second messengers, including cAMP, Ca^2+^, and protein kinase C, increase. These messengers induce the expression and activity of NAT and HIOMT (Figure 2). The last step is the rate-limiting step in the biosynthesis of melatonin. Once produced, melatonin is rapidly diffused into the adjacent capillaries and possibly into the third ventricle of the brain [11,24].

**Figure 3:**
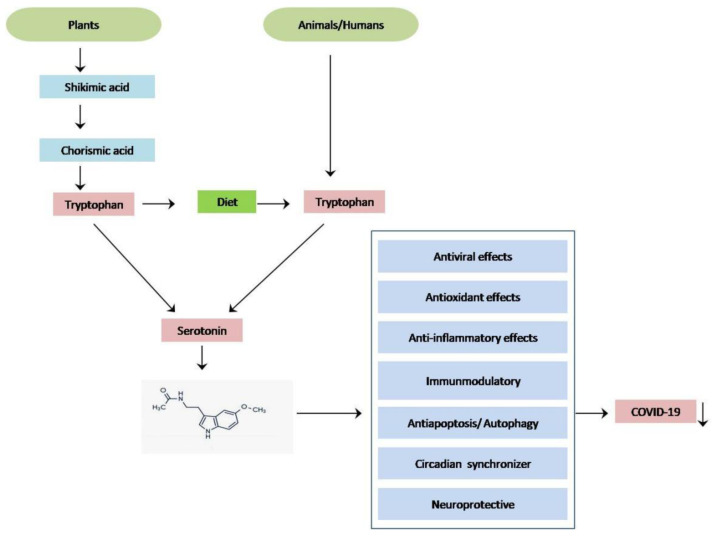
Biosynthesis of melatonin from plant sources and the potential reason to use melatonin as a natural supplement instead of drugs for preventing coronavirus infection.

Melatonin also exists in some types of seaweed and in different portions of many edible plants, where it is involved in plant functions including growth and development, acting as an auxin-like molecule [10,25]. Evidence suggests that melatonin can perform as a signaling molecule in plants during biotic and abiotic stress, respond to plant defense mechanisms against pathogens, and stimulate stress tolerance activity under adverse conditions [25]. Melatonin synthesis is initiated in plants by tryptophan, which is usually metabolized de novo through the specific shikimate pathway (Figure 3). There are seven different steps of biosynthesis to convert shikimic acid to chorismate, the precursor of tryptophan. It has been hypothesized that the sites of mitochondria and chloroplasts in plant cells produced higher amounts of melatonin than animal cells [26]. Several reviews reported that many foods containing melatonin, non-processed food, and fermented food show antioxidant activity, which can boost human health. Recently, a systematic review documented the ability of food sources of melatonin to improve sleep quality [10,27,28,29,30]. Since it is well documented that melatonin has potential physiological and biological benefits in both plants and humans [24], we can hypothesize that the intake of plants or animals containing melatonin supplements might reduce the complications of COVID-19 disease, as depicted in Figure 3.

It is well described that melatonin is essential for the control of several physiological processes, including the elimination of free radicals and the stimulation of the immune system through activation receptors (i.e., MT1 and MT2) [31]. The possibility of melatonin as a preventative and adjuvant therapeutic medication for the Omicron variant of COVID-19 could be considered. In this paper, we hypothesize melatonin’s (a) antiviral, (b) anti-inflammatory, (c) immunoregulatory, (d) antioxidant, (e) antifibrotic, (f) anti-apoptotic, (g) chronobiotic, and (h) neuroprotective properties. Melatonin’s unique multifactorial therapeutic potential could make it a more effective medication and adjuvant therapy for COVID-19 disease than other therapeutic interventions.

## 2. Melatonin as an Anti-Viral Agent

**Figure 4:**
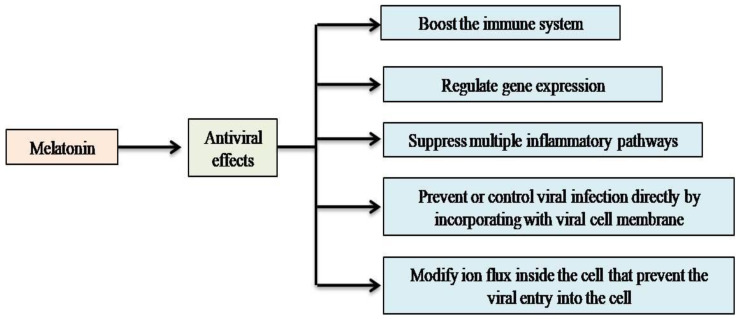
The effects of melatonin as a virus fighter ofCOVID-19 disease.

Melatonin exhibits effective inhibitory effects against viral infections in humans [32]. There is a lack of research on the potential effects of melatonin in the case of the newly discovered coronavirus. Previous studies have reported that melatonin attenuates the encephalomyocarditis virus causing severe inflammation in the nervous tissue, and reduces the high mortality rate [33]. It has also been reported that melatonin prevents Ebola patients from developing hemorrhagic shock syndrome [34]. The immunotherapeutic treatment of HPV-related tumors was more effective when melatonin adjuvant with indoleamine 2,3-dioxygenase-1 inhibitor was administered [35]. Previous research has shown that melatonin’s antioxidant properties can interact directly with the SARS-CoV-2 membrane and its genetic material [36]. A potent antioxidant, melatonin is a tiny amphiphilic molecule that can rapidly traverse membranes and penetrate all cells and organelles without restriction [37]. Evidence indicates that melatonin has a pleiotropy mechanism that regulates gene expression [38]. Based on this finding, molecular research is required to elucidate the biological activity of melatonin in COVID-19 and its variants, as outlined in Figure 4.

Even though researchers are still looking into melatonin’s direct antiviral effects, studies have shown that it can stop and control the spread of animal coronaviruses between species. One report found that melatonin stopped the coronavirus from infecting animals and stopped it from spreading. This was the first study to show that melatonin may fight viruses directly [39]. 

## 3. Anti-Oxidative Effects of Melatonin 

Several risk factors are associated with COVID-19 disease severity [40]. Most of these factors are related to oxidative stress [41]. Oxidative stress worsens the disease, and antioxidants could make it less severe [42]. Cytokine storm is a common COVID-19 symptom and a major cause of oxidative stress [43].

Several randomized clinical trials and adjuvant therapies haveobserved the effects of antioxidants, including melatonin, on SARS-CoV-2, but there are few experiments on Omicron variants (B.529) [44,45,46,47]. A cross-sectional clinical trial experiment and adjuvant therapy found that the dose of 3 to 10 mg of melatonin has preventative and therapeutic effects for COVID-19 disease, but it must be further evaluated, especially in children, and the efficacy of melatonin against the Omicron variant requires investigation [45,48]. Recently, a mini review hypothesized, according to an experiment, that melatonin with REGN-CoV-2 adjuvant therapy might significantly prevent the infection of Omicron variant in immunocompromised and elderly patients [49].

Given melatonin’s antioxidant properties, previous experiments with viruses, and recent clinical trials, it is hypothesized that melatonin might protect organs and tissues from oxidative stress because of its antioxidant properties and signal modulating effects [50]. The efficacy and tolerability of high-dose melatonin for COVID-19 pneumonia patients have been reported, and no side effects were observed, except drowsiness [51]. According to a clinical evaluation, the only medication that was significantly connected with frequent positiveoutcomes was melatonin [52]. Melatonin offers better defense against oxidative damage and free radicals than conventional vitamins C, E, and Trolox, as has been abundantly demonstrated [53]. 

Generally, melatonin exerts it antioxidant effects by (1) direct scavenging; (2) metabolizing compounds with high antioxidant activity, known as “melatonin antioxidant cascade”; (3) stimulating the synthesis of antioxidant enzymes while suppressing pro-antioxidant enzymes and pro-inflammatory enzymes; and (4) stabilizing the mitochondrial inner membrane integrity, thus reducing electron leakage and ROS generation, as well as maintaining mitochondrial homeostasis [53,54]. Additionally, it controls the receptor-independent action [24] and dependent gene expression of the antioxidant enzyme [55]. The benefits of melatonin as an antioxidant, cell protector, and potential disease preventive have been thoroughly explored in a number of articles [56]. Controlling gene expression by melatonin was initially suggested by Menendez-Pelaez et al. [57]. The expression and regulation of numerous antioxidative enzyme-related genes have been thoroughly described [58].

**Figure 5:**
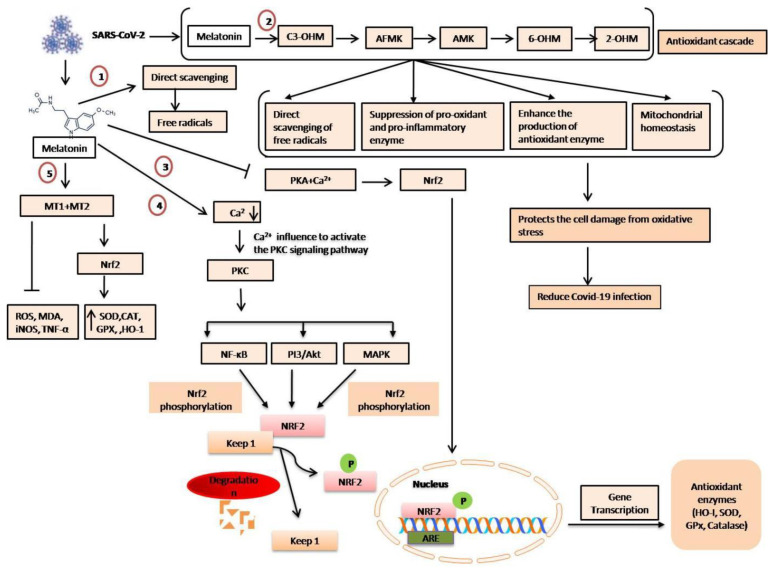
Hypothetical pathways of melatonin and its metabolites in the treatment of COVID-19. (1) Melatonin has a direct free radical scavenging capacity. However, this effect is not receptor-dependent. (2) The metabolites of melatonin protect the COVID-19-infected cells from oxidative damage byexhibiting strong antioxidant effects. (3) Melatonin inhibits protein kinase A (PKA) and Ca^2+^ signaling that could modulate gene transcription regulation and antioxidant enzyme concentration. (4) Melatonin may lower Ca^2+^ concentration, which can be influenced to regulate various mitogen protein kinases related to the PKC-mediated signaling pathway, i.e., extracellular signal-regulated kinase (ERK) and Jun N-terminal kinase (JNK), NF-κB, or PI3/Akt pathway activation, and thus modulate gene transcription. (5) Binding with MT1/MT2, melatonin activates the Nrf2 signaling pathway and inhibits free radicals, nitric oxide synthases, inflammatory cytokines, and oxidative stress.

Among the melatonin metabolites, cyclic 3-hydroxymelatonin (C3-OHM), N1-acetyl-N2-formyl-5-methoxykynuramine (AFMK), N1-acetyl-5-methoxykynuramine (AMK), 6-hydroxymelatonin (6-OHM), and 2-hydroxymelatonin (2-OHM) are most known for their high antioxidant properties [54,59]. Several reports have been published affirming that the antioxidant efficiency of melatonin metabolites for scavenging ROS and preventing protein oxidation is much higher than melatonin [60]. Therefore, it seems that at least in general, their protective activities against oxidative stress follow the order AMK > melatonin>AFMK. Research has also shown that for the reaction with the peroxyl radical, c3-OHM was faster than melatonin, AFMK, and AMK, as well as 100-fold faster than water-soluble vitamin E (Trolox) [60,61,62]. Based on the analysis of several experiments and reviews, we can hypothesize that melatonin and its metabolites might be an effective COVID-19 treatment, as detailedin Figure 5.

Melatonin’s high antioxidant activity works in tandem with its anti-inflammatory effects by up-regulating antioxidative enzymes (e.g., superoxide dismutase). It may also interact directly with free radicals while simultaneously acting as a free radical scavenger [63]. The production of low-density oxidized protein activates innate immune response by the overproduction of IL-6 alveolar macrophages via TLR4/NF-κB signaling [34]. TLR4 is an innate immune system receptor that can also be a melatonin therapeutic target. Melatonin has also shown anti-inflammatory properties via TLR4 signaling in brain ischemia, gastritis, and periodontitis disease models [64]. Melatonin’s anti-oxidative impact has also been demonstrated in ALI caused by radiation, sepsis, and ischemia reperfusion [65]. Severe inflammation, hypoxemia, and mechanical ventilation with high oxygen concentrations necessarily promote oxidant production locally and systematically in patients with ALI/ARDS, especially when the disease is developing, and in patients treated in intensive care units (ICUs) [66]. Accordingly, we speculate that excessive oxidation is also likely involved in COVID-19. In extensive studies by Gitto et al. [67], melatonin’s antioxidant and anti-inflammatory effects on newborns with respiratory distress in the lung have been successfully documented. We thus speculate that using melatonin to decrease inflammation and oxidation in people infected with coronaviruses would be advantageous.

According to studies, melatonin prevents oxidative stress from causing neutrophil apoptosis and restores redox equilibrium by increasing the synthesis of antioxidants [68]. Melatonin has also been demonstrated to restore activities of neutrophils such as phagocytosis, GSH, and GR. Neutrophils are the first line of defense against pathogens, and their over-activation is one of the main reasons why COVID-19 induces cytokine storm [69]. Because of its strong antioxidant properties and ability to stimulate the redox system, melatonin could play a role in reducing coronavirus infection. Melatonin has a short half-life, and its circulatory concentration may not be sufficient to fight oxidative stress [66]. Moreover, melatonin production decreases withage, and certain diseases also alter the circadian system [70]. As a result, melatonin could be administered to fight against oxidative stress and linked clinical consequences. Exogenous melatonin treatment has already been shown to improve immune cell protection by optimizing redox balance [71].

## 4. Melatonin’s Anti-Inflammatory Effects

**Figure 6:**
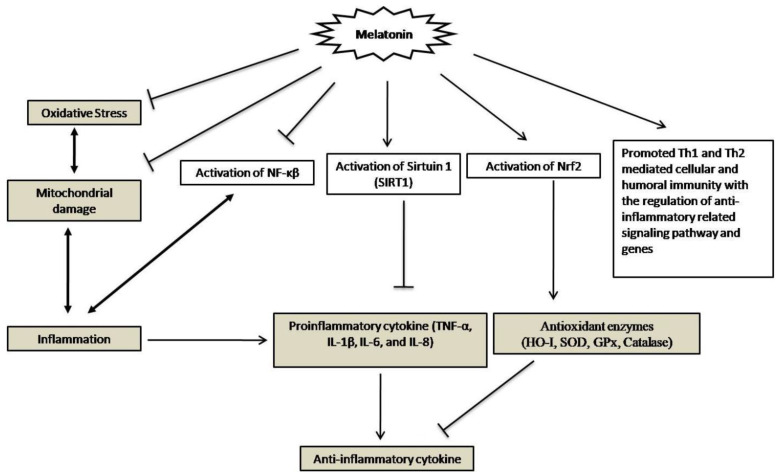
Possible anti-inflammatory effects of melatonin on COVID-19 infection.

Cytokine storm is the crucial factor in initiating COVID-19 viral inflammation and is related to other complications. The severity of COVID-19 decreases the circulating B cells, CD8^+^ cells, CD4^+^ cells, and NK cells, and also eosinophils, monocytes, and basophils. Melatonin has a well-researched, potent anti-inflammatory action [72]. Melatonin has reportedly been shown to reduce lung damage and inflammation via sirtuin-1 (SIRT1) pathways. Melatonin’s anti-inflammatory properties may be mediated via SIRT1 through the inhibition of the high mobility group box 1(HMGB1) protein or the down-regulation of NF-κB activation [73]. Experiments suggest that melatonin can exert its anti-inflammatory effects by modulating inflammatory cytokine pathways [74]. The presence of melatonin receptors in a mast cell line modulates an anti-inflammatory pathway via inhibition of TNF-α release [75] and prevents inflammatory processes by scavenging free radicals and activating endogenous antioxidant enzymes [76]. Under severe inflammatory circumstances, melatonin is typically effective in protecting cells from oxidative damage. One study discovered that melatonin adjuvant therapy helped patients with COVID-19 disease by reducing inflammatory cytokines and improving disease control [77]. Additionally, the researchers stated that melatonin therapy at a dose of 9 mg per day for 14 days affected the expression of genes linked to the humoral and cellular immune systems Th1 and Th2 receptors [78]. The summarized studies and reviews support that melatonin could be an effective anti-inflammatory agent againstCOVID-19 infection, as described in Figure 6.

The action of melatonin in the gut after exogenous administration through a capsule, tablet, supplement, or food requires further study. Melatonin is an important regulator in reducing gut inflammation and maintaining gastrointestinal tract homeostasis, as well as controlling the circadian cycle [79,80]. Inflammation and elevated cytokinelevels are among the major complications of COVID-19 infection [5]. Inflammation and elevated cytokine levels trigger the imbalance of the gut immune system as well as gut homeostasis, known as post-acute COVID-19 syndrome (PACS) [81]. The expression of angiotensin-converting enzyme-2 on the enterocytes and colonocytes is gradually increased due to the epithelial invasion of SARS-CoV-2. As a result, angiotensin-converting enzyme-2 protein is down-regulated and promoted to develop the molecular mechanism of severe acute respiratory syndrome and systemic inflammatory response with this coronavirus [81]. Intestinal microbial dysbiosis has also been associated with acute SARS-CoV-2 infection and PACS. Long-term respiratory dysfunction after COVID-19 is reported to alter the intestinal microbiota and continuously elevate lipopolysaccharide-binding protein levels [81,82]. Studies showed that dysbiosis is most common in hospitalized COVID-19 patients [82]. Interestingly, studies have found that patients who experienced GI symptoms with COVID-19 infection were more likely to have a more severe COVID-19 illness, with a greater need for intensive care unit admission and ventilation [83,84].

There are several in vitro and animal experiments and limited studies in humans suggesting that supplemental melatonin may have an ameliorative effect on colitis [79] and improve inflammatory bowel disease (IBD) [85] and irritable bowel syndrome (IBS) [86]. Studies show that daily supplementation with melatonin has a variety of beneficial effects over the long term [87,88,89]. It is also well documented from our previous discussion that melatonin and its metabolites directly scavenge free radicals and activate antioxidant enzymes [90,91]. In animal studies, melatonin treatment normalized the aging-related oxidative stress and inflammatory signs (COX-2, NF-κB) and pro-apoptotic enzymes (caspase-3 and -9) and improved the age-related changes in the gallbladder, pancreas, and other smooth muscle functions [87,92,93,94]. Several in vitro and animal studies have reported that melatonin regulates the extensive gut immune system and has important general anti-inflammatory and immunomodulatory effects by increasing the IL-10 production and inhibiting the production of IFN-γ, TNF-α, IL-6, and NO, suggesting that melatonin may exert benefits in ulcerative colitis (UC) [95,96,97,98,99]. 

**Figure 7:**
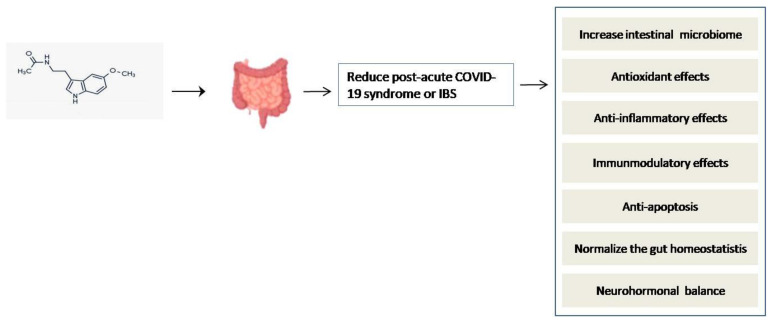
Administration of melatonin can enhance gut homeostasis and reduce post-acute COVID-19 syndrome.

Melatonin may also influence the gastrointestinal (GI) tract indirectly, through the central nervous system and the mucosa, by receptor-independent scavenging of free radicals serving to reduce inflammation and hydrochloric acid. It can also help to stimulate the immune system, regenerate the epithelial tissue, and enhance microcirculation and neuronal hormone balance [100]. According to the above discussion and experimental reports, we can hypothesize that the administration of melatonin could be an excellent preventative agent against COVID-19 infection, as shown in Figure 7.

## 5. Immunomodulatory Effects of Melatonin

The coronavirus infects the epithelial cells of the respiratory tract and dendritic cells and presentsantigens to T cells. CD8^+^ T cells release pro-inflammatory cytokines which induce cell apoptosis [101]. Both the infection and cell death influence the immune response. In this instance, the membrane-bound MT1 and MT2 receptors of melatonin, members of the superfamily of G-protein-coupled receptors, can act physiologically [102]. Measuring the quantity of cyclic adenosine monophosphate revealed that melatonin had both cellular and humoral immunological effects in both animals and humans (cAMP) [103]. The over-activation of neutrophils, lymphocytes, and CD8+ T cells in blood is the major clinical feature of COVID-19 [104]. By promoting the growth and maturation of NK cells, T and B lymphocytes, granulocytes, and monocytes, melatonin can improve the immunological response [105]. 

**Figure 8:**
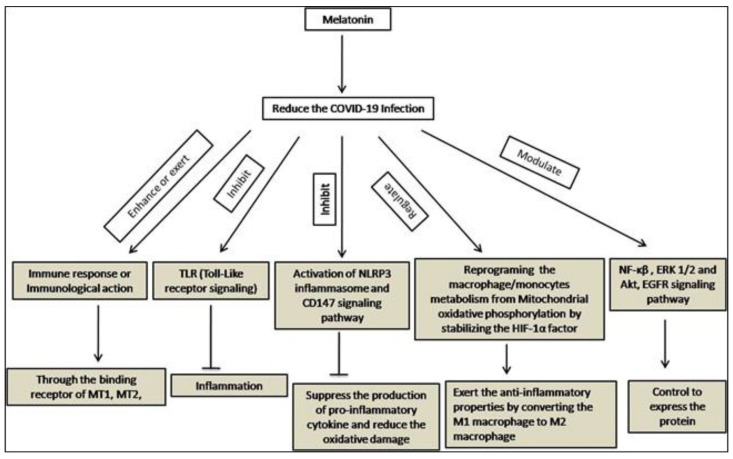
Immunomodulatory effects of melatonin.

The NOD-like receptor 3 (NLRP3) is associated with lung diseases caused by influenza A virus, syncytial virus, and bacterial infection [106]. Melatonin’s effectiveness in controlling NLRP3 has been demonstrated in radiation-induced lung damage [107]. Studies have reported that SARS-CoV-2 can invade the host cells through CD147 S protein [108]. The interactions between melatonin and CD147 S protein have not been directly studied. However, in the instance of AngII-induced ventricular hypertrophy, the antioxidant action of melatonin has been demonstrated through blocking the CD147 signaling pathway [109]. Toll-like receptors (TLRs) are the primary target of many respiratory viruses [110]. In addition, TLR pathways have crucial roles in the immunopathogenesis of several viral diseases [111]. Numerous studies have demonstrated that melatonin inhibits TLR signaling pathways and may have therapeutic benefits for the management of inflammatory illnesses [112]. From this perspective, melatonin may be crucial for patients with SARS-CoV-2 infection who receive immunotherapy (Figure 8).

**Figure 9:**
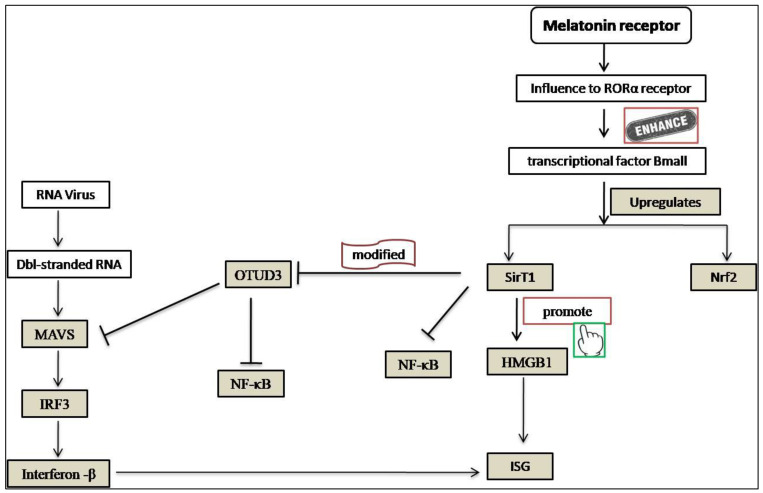
Molecular mechanisms of melatonin in the modulation of the SARS-CoV-2 virus-mediated signaling pathway to induce interferon and interferon-stimulated genes.

SARS-CoV-2 virus-infected monocytes/macrophages reprogram their glycolysis metabolism for ATP production via the generation of ROS that stabilizes the hypoxia-inducible factor-1α (HIF-1α) [113]. This metabolic profile generates more cytokines which destroy alveolar lining cells, cause T cells to die, and worsen coronavirus infection. Melatonin uses mitochondrial oxidative phosphorylation to change pro-inflammatory glycolytic M1 into anti-inflammatory M2 macrophages [114]. This effect of melatonin may be exerted through the down-regulation of HIF-1α [115]. However, it was also found that melatonin affected the protein expression level of HIF-1α and NF-κB in a murine model of hypoxic pulmonary hypertension [116]. Melatonin could minimize pulmonary artery smooth muscle cell proliferation and the phosphorylation levels of Akt and extracellular signal-regulated kinases 1/2 [63] (Figure 8). A recent study revealed that melatonin stimulated the silent information regulator 1 (SIRT1), which may increase the effectiveness of type I interferons in combating viruses [117]. Additionally, it was observed that, in addition to altering the virus-mediated signaling pathway, the damage-associated molecular pattern protein HMGB1 was also prevented from possibly influencing interferon-stimulated genes (ISG). The chemical mechanism behind melatonin’s effectiveness as an antiviral is briefly depicted in Figure 9.

## 6. Anti-Fibrotic Effect of Melatonin

Another complication of COVID-19 disease, pulmonary fibrosis, is characterized by a continuous decrease in lung function as a result of irreversible scarring of lung tissue [118]. Age, smoking, drug exposure, and genetic predisposition are also pulmonary fibrosis factors [119]. SARS-CoV-2is the most predominant factor for lung fibrosis [120]; therefore, we sought to clarify the potential role of melatonin in preventing fibrosis. 

**Figure 10:**
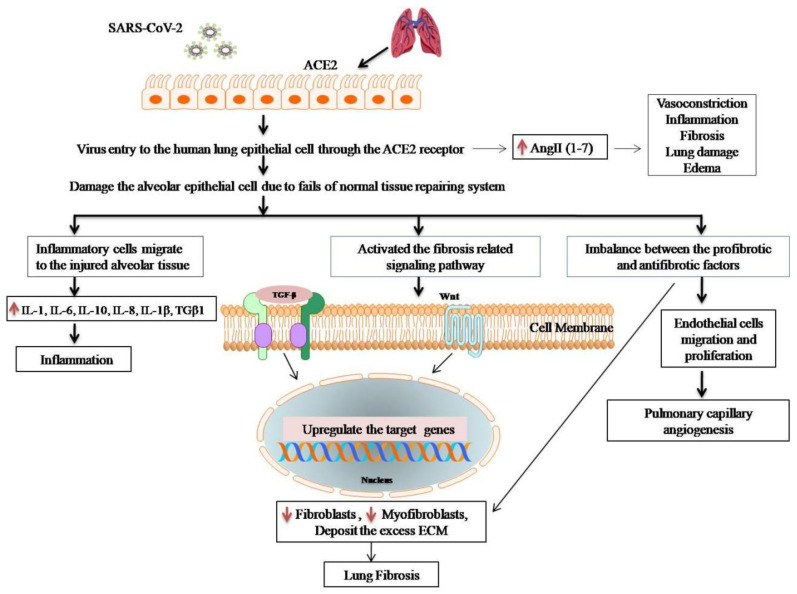
Summary of lung fibrosis mechanisms by the SARS-CoV-2 virus.

Through the ACE2 receptor, the coronavirus enters and damages human lung cells, producing angiotensin (1–7), whichcauses vasodilatation, inflammation, edema, and lung fibrosis. The natural tissue repair process is hampered by the impairment of AEC2 receptors [118]. Several growth factors and cytokines, including monocyte-1, chemo attractant protein (MCP-1), transforming growth factor β1 (TGF-β1), TNF-α, fibroblast growth factor (FGF), platelet-derived growth factor (PDGF), IL-1β, and IL-6, are over-expressed and released from the coronavirus-infected cells [121]. Due to the dysfunction of vascular tissue, this progresses to fibrosis and causes myofibroblasts [122]. Extracellular matrix (ECM) build-up in interstitial tissues and basement membranes is caused by myofibroblasts, and this eventually results in the loss of alveolar function [121]. The fibrosis formation by the coronavirus is shown in Figure 10.

**Figure 11:**
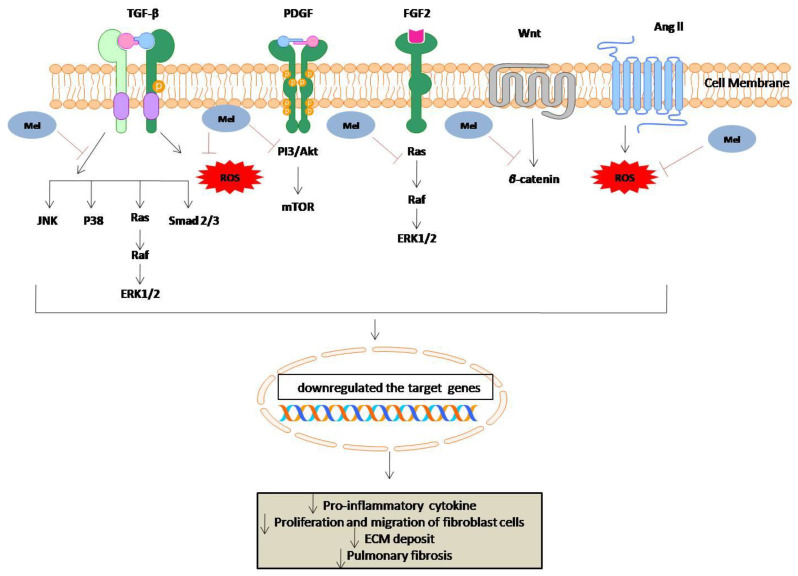
The potential effects of melatonin on pulmonary fibrosis.

Melatonin can play an antifibrotic role with the inhibition of oxidative stress [123]. Oxidative stress is the most crucial mechanism for the development of fibrosis [124]. Melatonin might be a suitable option to prevent oxidative stress because it is a potent antioxidant. Measuring indirect fibrosis markers, such as aspartate aminotransferase (AST), alanine aminotransferase (ALT), alkaline phosphatase (AP), and pro-inflammatory cytokines, can help identify the antifibrotic effects of melatonin: IL-6, IL-1β, TNF-α, TGFβ, and PDGF. With ideal antioxidant properties, melatonin not only removes the ROS but also activates the endogenous antioxidant enzymes [125,126]. Based on the literature review, the pathophysiology of pulmonary fibrosis and the defensive effects of melatonin are briefly hypothesized in Figure 11.

**Figure 12:**
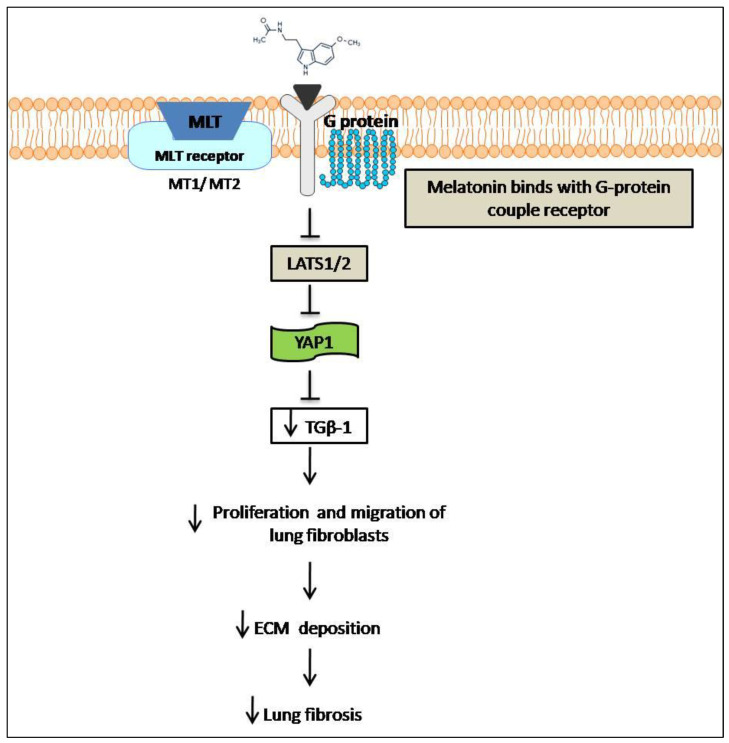
Anti-fibrosis effects of melatonin through the Hippo/YAP signaling pathway.

Studies have shown that the Hippo signaling pathway is responsible for various pathological processes [127]. Yes-associated protein (YAP), known as a key downstream effector of Hippo, has attracted interest in the study of human diseases. Zhang et al. reported that oroxylin A elevated angiogenesis in liver fibrosis by inhibiting Hippo-YAP signaling [128]. Because GPCR signals regulate the Hippo signaling pathway and since melatonin activation is mostly dependent on MT1 and MT2 [129], we can conclude that MT1 and MT2, which are known as classical GPCRs, may activate the Hippo signal cascade. Experiments demonstrate that melatonin can also have an anti-fibrotic effect during the course of idiopathic pulmonary fibrosis (IPF) by inhibiting TGF-β1 [127] (Figure 12).

## 7. The Anti-Apoptotic Effects of Melatonin

**Figure 13:**
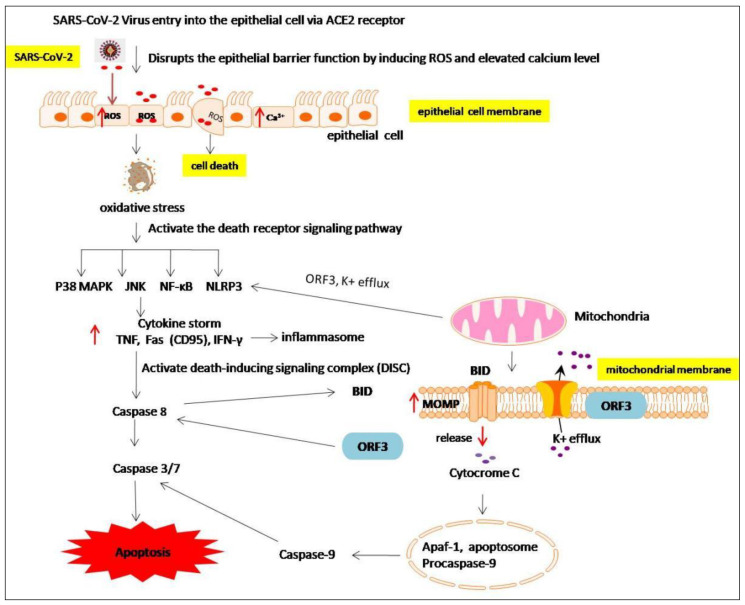
Possible mechanisms of cell death or apoptosis induced by SARS-CoV-2 proteins and cytokine production.

SARS-CoV-2 infection also affects the cell apoptosis cycle [130]. During the infection process, viruses enter lymphocytes via the ACE2 receptor, causing cellular oxidative stress and increasing the cellular Ca^2+^ level, resulting in immune system hyperactivation. Inflammasomes form as a result of the activation of inflammatory pathways such as NF-κBand NLRP3. Finally, a death-inducing signaling complex (DISC) containing the adaptor protein procaspase-8 is formed [131]. Caspase-8 cleaves caspase-3/7 directly or processes the BH3-related member of BID, which is moved into the mitochondria and triggers mitochondrial outer membrane permeabilization (MOMP). Dysregulation of mitochondrial membrane permeabilization eventually leads to cell death via cytochrome C (cyt c) release and activating factor-1 (Apaf-1)/caspase-9 apoptosome [131] (Figure 13). The apoptotic pathway is also triggered by the raised ROS and K^+^ efflux that facilitates the release of the ORF3 viroporin and causes the formation of NLRP3 inflammasomes [132]. ORF3a is also responsible for the release of the virus [133]. The ablation of this protein in animal models has been found to limit viral propagation [134]. Experiments on various cell lines revealed that ORF3a activates caspase-8 [135], ultimately leading to apoptosis. 

Based on the findings of various researchers, it is possible to speculate that melatonin may have anti-apoptotic effects on SARS-CoV-2. Experimental results reveal that melatonin reduces caspase-3, -8, and -9 activities in acute liver failure caused by a hemorrhagic virus [136]. It has also been reported that it could suppress apoptosis by the down-regulation of Mst1-Hippo signaling in virus-induced myocarditis [137]. Melatonin boosts the anti-apoptotic proteins Bcl-2 and Bcl-xl while suppressing Bax and cytosolic cyt c release produced by viral infection [138]. Studies have proved that it can also decrease brain apoptosis as well as increase the survival rate in Venezuelan equine encephalitis [139]. Based on theliterature review, the anti-apoptotic effects of melatonin on virus-infected cells are well documented [140], but its role in the context of COVID-19 is still unclear. The probable anti-apoptotic effects of melatonin are hypothesized inFigure 14.

It is well documented that the primary sites of melatonin synthesis are mitochondria, which could therefore be relevant players ininhibiting the cellular apoptosis induced by coronavirus infection [141]. Mitochondrial dysfunction and overproduction of ROS are the most prominent factors that induce liver injury in SARS-CoV-2 infection. The most effective melatonin receptors, MT1 and MT2, are seven transmembrane-spanning proteins from the GPCR superfamily that have high affinity for binding and can be triggered even at low melatonin concentrations in the cell [142]. In a previous study, Zhang et al. showed that treatment with melatonin prevents lung injury by regulating the apelin-13 receptors, also known as GPCR [143]. Under stress conditions, it could reduce the cellular Ca^2+^ influx by binding to the intracellular CaM proteins, which act as intracellular secondary messengers, as well as regulate the cAMP-mediated signaling pathway [144]. Melatonin can also modulate the stress-induced mitochondrial permeability transition pore (mPTP) and optimize the mitochondrial oxidative phosphorylation (OXPHOS) that prevents the release of cytc and cardiolipin peroxidation through the inhibition of mPTP [145]. Melatonin can control redox homeostasis and endoplasmic reticular stress by exhibiting excellent antioxidative effects that reduce mitochondrial damage and protect cells from apoptosis [39]. More research is needed to clarify this hypothesis and pave the way for future drug development.

**Figure 15:**
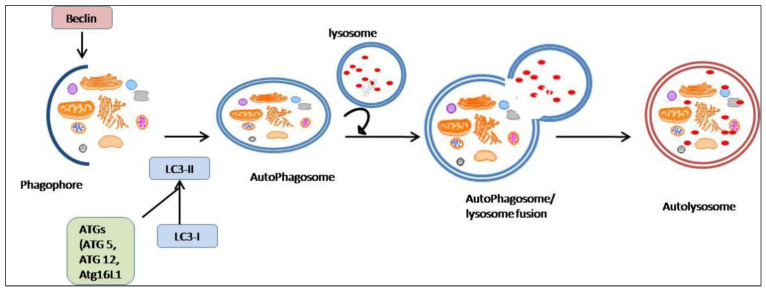
Autophagy process. Autophagy begins with the development of a phagophore associated with the autophagy protein Beclin 1, followed by autophagosome production and the attachment of LC3-II to the membrane. The fusion of autophagosomes and lysosomes results in the release of degraded material into the cytoplasm, which causes proteolysis.

Coronaviruses can also hijack the cellular machinery process to complete their replication. Autophagy is a self-destructive process that involves the removal of dysfunctional or unfolded or misfolded proteins and degrades the malfunctioning cellular components, including proteins, damaged organelles, and invasive microbes, through the four steps of the autophagy process. The first stage of the autophagy process is the isolation of the membrane, followed by the formation ofa “phagophore”, which is a double membrane. The phagophore engulfs the substrates and sequestrates them within an autophagosome [146]. The mature autophagosome merges with a lysosome and generates an autolysosome, asshown in Figure 15. Several proteins, autophagy factors, and genes are involved in the autophagy process, including Beclin 1, LC3-I and-II (light chain), and ATGs (genes related to autophagy) [147]. 

**Figure 16:**
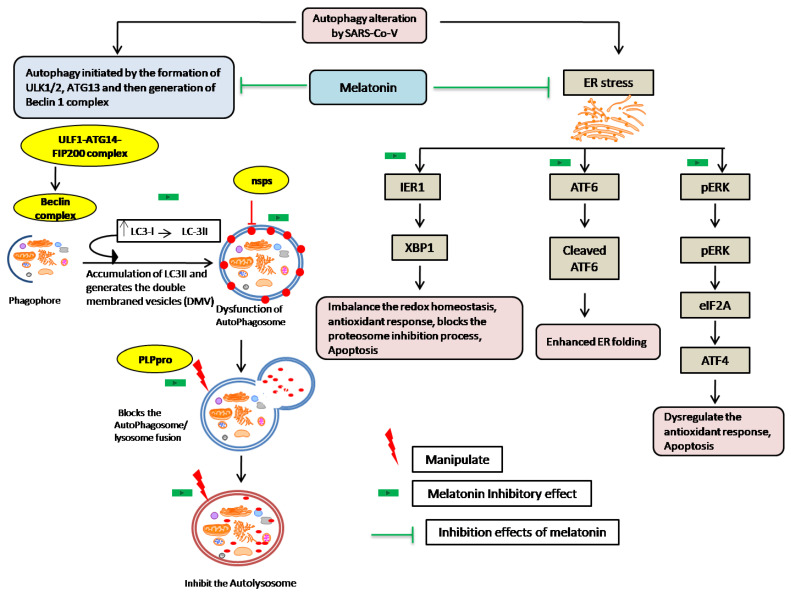
Autophagy regulation by SARS-CoV-2 and melatonin’s ameliorating effects.

SARS-CoV-19 infection triggers several processes, such as a reaction with host cell surface receptors and autophagic adaptors, along with oxidative and ER stress induction [148]. Coronaviruses play a hijacker role during the general process of autophagy and modulate autophagosomes through the recruitment of host autophagosomal protein and use it as their replication material for survival in the cell [149]. The SARS virus attaches to epithelial cells via the ACE2 receptor and enters the cell via ACE2-mediated endocytosis during the infection phase. The virus membrane and the endosome membrane then join forces to allow the viral genome to enter the cell. The genome contains several open reading frames (ORFs) that are localized to the endoplasmic reticulum and encode essential proteins. These encoded proteins are translated into ORF-1a and ORF-1b proteins, which are large polypeptide coding genes. The proteins are then broken into 16 nonstructural proteins (nsps) of the virus by papain-like protease (PLPro), which inhibits autophagosome maturation and autolysosome formation [150]. An evolutionary analysis of the SARS-CoV-2 genome sequences of 351 clinical samples revealed mutations in NSP6, a protein that has an inducing effect on autophagosome formation [151]. In general, autophagy is triggered by the creation of the ULK1/2-ATG13-FIP200 complex, followed by the generation of the Beclin1 complex and LC3 mediators, resulting in the formation of an autophagy vesicle. Coronavirus proteins can regulate autophagy by modulating autophagy regulatory proteins at different phases of autophagy. Viral infection can impair ER function, as well as cause ER stress and cell death. Induction of ER stress activates the inositol-requiring enzyme (IRE1), activating transcription factor ATF6, and protein kinase RNA-like ER kinase (PERK). The activation of enzymes or proteins causes either ER homeostasis or apoptosis as an antiviral response, as illustrated in Figure 16.

Melatonin can modify and regulate autophagy via its decisive antioxidative actions, which play a role in suppressing ER stress generated by viral infections [144]. Regarding viral infections, melatonin treatment has been shown to inhibit rabbit hemorrhagic disease virus (RHDV)-induced autophagy in rabbit hepatocytes [148]. Melatonin injection controls autophagy by increasing autophagosome proteins such as Beclin1, developing a complex protein with Atg16L1 and Atg5, and constructing an autophagosome membrane. Melatonin can boost senescence-induced autophagy in neurons via SIRT1 deacetylation of the NF-B RelA/p65 subunit [149]. According to a recent publication (Table 1), we can hypothesize the effect of melatonin on autophagy in the case of coronavirus infections, and the controlling method is depicted in Figure 16. However, a more detailed investigation is required.

## 8. Melatonin as a Chronobiotic Agent

Among several other complications, sleep deprivation and insomnia are documented in COVID-19 patients, especially among the aging population, who are more vulnerable to suffering from coronavirus infection. The main reasons for this complication include age, lower immunity, and hormonal imbalance. Several factors are considered to be responsible for this complication, including higher psychological stress and depression due to forced lockdown and social isolation, reduced melatonin levels with age, impaired immunity, inadequate exposure of individuals to light in the evening, and disturbed circadian rhythm, which are all responsible for altering general immunological function [1]. It is also reported that increased stress and depression in socially isolated aged people lead to increased pro-inflammatory and decreased antiviral immune responses [156]. Dysregulation of circadian systems is considered the cause of other diseases, including cardiovascular, neurodegenerative, and metabolic syndromes [157]. Generally, sleep and circadian rhythm play primary roles in maintaining physiological function, as well as the regulation of hormonal metabolism. This dysregulation is associated with severe complications such as obesity, insulin insensitivity, diabetes, impaired glucose, lipid homeostasis, reversed melatonin and cortisol rhythms, and abnormal appetite [158]. Recently, a GIS analysis investigation found that insomnia is frequently present in COVID-19 depending on regional variability, along with other multifactorial determinants [159]. Another cross-sectional study in France reported that young people aged 18 to 34 years suffer slightly worse sleep problems more frequently than elderly people [160]. This might be due to overusing mobile or other electronic devices that greatly affect the sleeping cycle, as it has been reported that pineal melatonin is disrupted due to exposure to electromagnetic radiation [161]. Melatonin is a pivotal chronobiotic agent that plays a major role in the circadian cycle. As previously documented, it not only regulates the circadian cycle but has wide implications in cellular proteins and enzymes, as well as many biological and physiological benefits, including innate and adaptive immunity [162].

**Figure 17:**
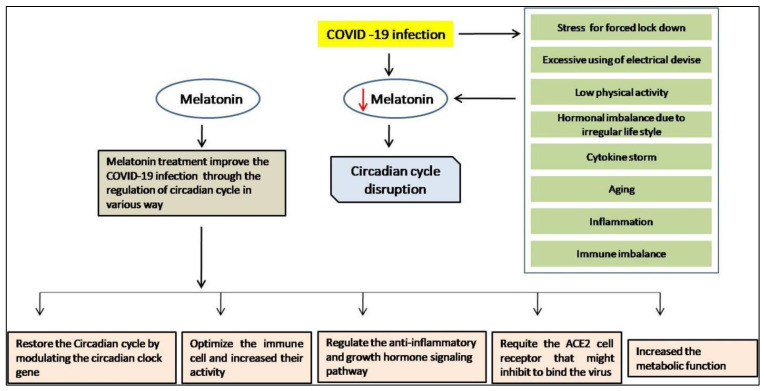
The roles of melatonin in restoring the circadian cycle.

According to several reports based on clinical trials and research articles, it could be hypothesized that melatonin may have the ability to maintain circadian rhythmicity in different ways, such as (1) by restoring the sleep cycle by modulating the gene of circadian rhythm in COVID-19 patients, along with elderly people; (2) improving physiological homeostasis, metabolism, and hormonal balance, which impact stress reduction and disease quality; (3) reducing the cytokine storm during coronavirus infection by improving innate and adaptive immunity; (4) optimizing the immune cells and their proliferations such as macrophage, leukocytes, and NK cell activity; and (5) regulating anti-inflammatory and growth hormone signaling [163] (Figure 17).

## 9. The Neuroprotective Effects of Melatonin

Studies have shown that infection with SARS-CoV-2 is also linked to neurological symptoms that affect the nervous system [164]. Acute neurological disorders, anosmia, stroke, convulsions, encephalopathy/encephalitis, seizures, andGuillain–Barre syndrome (GBS) are found in COVID-19 patients.Approximately 36% of COVID-19 patients exhibit neurological symptoms in the central nervous system (CNS) and the peripheral nervous system (PNS). Evidence indicates that coronavirus infection has severe complications in the CNS [165]. Recently, stroke and spinal cord complications were observed due to neurochemical alterations [148]. CNS symptoms of COVID-19 neurological manifestations are encephalitis, meningitis, and GBS caused by the direct neurotropic action and virus entry into the CNS [166]. Neuroinflammatory proteins and antibodies are found to fight against coronavirus infection through the observation of cerebrospinal fluid (CSF) [167]. However, symptoms of the PNS are less severe and manifest as neuralgia, hypoplasia, hyposmia, and hypogeusia [165]. Recently, several articles briefly documented multiple neurodegenerative diseases such as Parkinson’s disease (PD), Alzheimer’s disease (AD), and multiple sclerosis (MS) with their responsible neuroinflammatory cytokines as well as proteins against COVID-19 [168].

**Figure 18:**
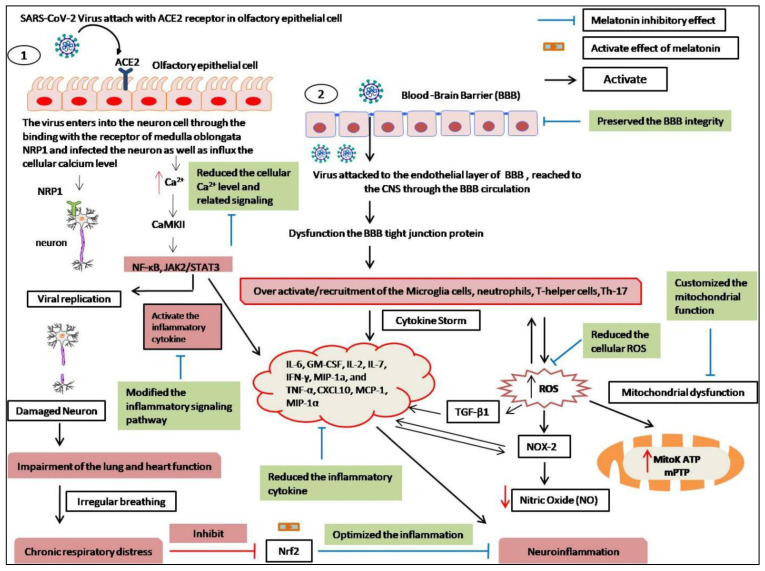
Neuroinflammationcaused by coronaviruses and possible outcomes of melatonin. 1. SARC-CoV-2 virus causes neuroinflammation through the direct entry of epithelial cell ACE2 receptor or 2. Destruction of blood-brain barrier in the central nervous. Melatonin can be reduced the neuroinflammation by elimination of infection as well as persevering the blood–brain barrier integrity.

The SARS-CoV-2 virus induces neuropathogenesis in two ways. First, it infects the brain through the direct penetration of the CNS by attaching to the ACE2 receptor of the olfactory epithelial bulbor the olfactory nerve (Figure 18) [169]. The main entry points of this virus are the nose, mouth, lymphatic or the hematogenous route, and finally, the olfactory bulb. The presence of the ACE2 receptor in the nasal mucosa and the brain contributes to its neuroinvasive nature. After binding with the ACE2receptor, cleaving of the spike (S) protein of SARS-CoV-2 by transmembrane serine protease 2 (TMPRSS2) facilitates the entry of the virus into cells [170]. Alternative receptors, including neuropilin-1 (NRP1), are also found at higher levels in the neuron cells of CNS as an entry receptor for SARS-CoV-2 [171]. Additionally, furin and cathepsin could allow the viral entry into the cells with low levels of TMPRSS2 expression [53]. Neuron cells are infected and then damaged by the virus. Viruses can infect the neuron cell through the peripheral nerve terminals or blood circulation, impair respiratory function, and modify the breathing pattern [172]. In addition, calcium influxes cause viral replication in neuron cells, which may alter Ca^2+^/calmodulin-dependent protein kinase II inflammatory cytokine responseNF-κBand JAK2/STAT3 signaling pathways. Calcium is necessary for viral entry, replication, and release into the host cell. Viruses induce an increased influx of the intracellular calcium level to facilitate their physiological action [173]. The second neuropathogenesis is the destruction of the blood–brain barrier (BBB) in the CNS [160]. SARS-CoV-2 attacks the endothelial layers of the BBB, which mainly functionto protect the CNS from a variety of toxins and microorganisms. The endothelial layers contain tight junction proteins that control the entry and exit of molecules from the blood toward the brain parenchyma [174]. SARS-CoV-2-infected cells have increased levels of several cytokines and chemokines, namely TNF-α, IFN-γ, interleukin-1 receptor antagonist (IL-1RA), IL-2, IL-6, IL-7, IL-8, IL-9, IL-10, and the colony-stimulating factor of granulocyte macrophages. In particular, COVID-19 patients with high levels of IL-6 have been observed to have a worse prognosis [175]. The high production and imbalance of all these molecules are essential factors in the breakdown of the BBB [174]. Microglial cell activation, increased cytokine production, and T-cell infiltration have been reported in post-mortem brain tissue [176]. Another possible neuronal damage mechanismis the redox imbalance in the cell [177]. Several reviews have reported that redox dysregulation is one of the typical reasons for acute COVID-19. Generally, the entry of virion into cells alters the immune cells’ function by increasing cellular and mitochondrial ROS production [178]. Among immune cells, monocytes and macrophages are primarily responsible for inflammatory reactions in coronavirus infection [179]. These immune cells can release a large amount of pro-inflammatory cytokines (IL-1β, IL-6, TNF-α, IL-8), which is typical in COVID-19 patients [180]. In addition, over-activation of neutrophils and decreasing lymphocyte levels are important factors in oxidative stress and COVID-19 severity [181]. It has also been documented that the reduction in lymphocytes, specifically T cells that are not hyporesponsive, can activate the ROS-mediated TGF-β1 during infection [182]. Several studies have reported that TGF-β is one of the major indicators of COVID-19 pathogenesis [183] and that blocking TGF-β could be a novel target to treat the infection [184]. Another major mechanism of neuroinflammation is the formation of ROS that activate the enzyme nicotinamide adenine dinucleotide phosphate (NADPH) oxidase (NOX) in COVID-19 [185]. NOX-2 activation is regulated by the binding of SARS-CoV-2 to the ACE2 receptor, which then reduces the bioavailability of ACE2 [186]. The reduction in the availability of ACE2 makes Ang II interact with AT1R, with the subsequent activation of NOX and the induction of oxidative stress and inflammatory responses [187]. NOX activation also reduces the bioavailability of NO, which leads to vasoconstriction, inflammation, redox imbalance, and endothelial dysfunction [181]. It can be activated by the release of TNF-α during the pro-inflammatory cytokine storm that causes local oxidative stress and endothelial dysfunction [188]. TNF-α-induced ROS production could contribute to the spread of COVID-19 symptoms to distant tissues such as the brain [109]. In addition, studies have hypothesized that excess cytosolic ROS produced by NOX can trigger the opening of the adenosine triphosphate (ATP)-sensitive mitochondrial potassium channel (mitoK ATP) and activate the permeability transition pore (mPTP), which causes the depolarization of the mitochondrial membrane and dysfunction. Mitochondria are the primary sources of ROS production in cells [189]. It is also reported that oxidative stress-induced mitochondrial dysfunction plays an important role in SARS-CoV-2viral infection, as well as inflammation [190]. In the brain, hypoxia causes mitochondrial dysfunction, which has also been found in patients with COVID-19 [191]. Several studies have shown that mitochondrial dysfunction increased pro-inflammatory cytokine production (CXCL-8, IL-6, CCL20, CCL3, CCL4, and IL-12) in the brain cells of COVID-19patients [192].

Since melatonin is a neurohormone and its metabolism and secretion originate mainly from the pineal gland in the brain [193], it could serve as a neuroprotective agent against coronavirus infection. It is not only secreted from the brain but also widely distributed in the retina, testes, ovary, placenta, glial cells, and lymphocytes [194]. The therapeutic potential role of melatonin against COVID-19 infection is still the subject of ongoing research [146], although numerous reports have already observed the effectiveness and outlined the possible doses of melatonin against SARS-CoV-2 [195]. 

The amphiphilic nature of melatonin, reaching all cellular organelles and binding to mitochondrial and cytoplasmic proteins, and its ability to spontaneously cross the BBB enhance its neural availability and therapeutic versatility in different ways [6,38] (Figure 18). Melatonin reduces the renin angiotensin receptor (RAS) with lower expression of ACE2 in the cell membrane, which may reduce the entry possibility of viruses into the neuron and glial cells [196]. According to research evidence, it can also be hypothesized that melatonin might block vascular endothelial damage [197]. Melatonin can rescue neuropathogenesis and preventneuronal damage from viral infection by the modulation of neuroinflammation [198]. 

Melatonin directly attaches to CaM with high affinity. This interaction can antagonize the binding of Ca^2+^, resulting in the modulation of the calcium/calmodulin-dependent kinase II (CaMKII) in axons and the progression of nerve regeneration [198]. Ca^2+^ is an intracellular secondary messenger to CaMinvolved in the regulation of inflammatory enzymes such as cyclic AMP (cAMP), CaM-kinase II, and NO synthase and the inflammatory signaling pathway, while melatonin inhibits the pathway and reduces inflammation by reducing the inflammatory cytokines [198,199,200,201]. Research has shown that it exerts a neuroprotective effect against radiation by the suppression of pro-inflammatory cytokines and the modulation of the GABA neurotransmitters [201]. GABA is a major neurotransmitter that can be reduced by a coronavirus infection-causing eclipse, neuroinflammation, and other neurodegenerative diseases [202]. Melatonin has also been shown to protect neurons against inflammation by activating the Nrf2 signaling pathway [203]. Recent research has proposed that effective doses of melatonin, about 100–300 mg, protect against the neurologic sequels of coronavirus infection [195].

## 10. Conclusions

**Figure 19:**
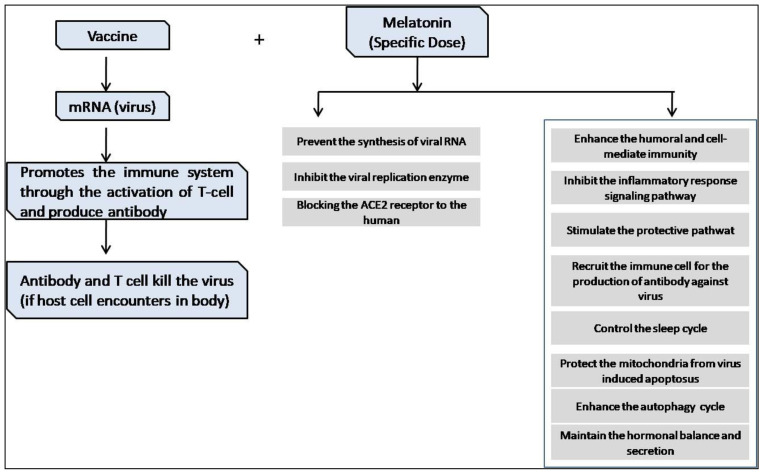
Future perspectives of the use of melatonin in vaccine adjuvant therapy.

Melatonin is an effective natural therapeutic drug against viral infections, according to our review of the literature and clinical experiments. General immunity is substantially compromised in COVID-19 disease, although melatonin can enhance humoral and cellular immunity. As a result, an appropriate dose of melatonin can alleviate the symptoms and severity of coronavirus infection. Furthermore, melatonin may serve as a potential immune booster in future vaccines that researchers have already clinically tested. Taken together, we can conclude that the use of melatonin in the appropriate amount would be a promising therapy option if paired with vaccines or other treatments that may increase the body’s natural immunity without interfering with the natural melatonin hormone. However, additional research and tests are required to clarify this notion. Figure 19 depicts a path that can help us better understand the use of melatonin in vaccine adjuvant therapy.

## Figures and Tables

**Figure 14:**
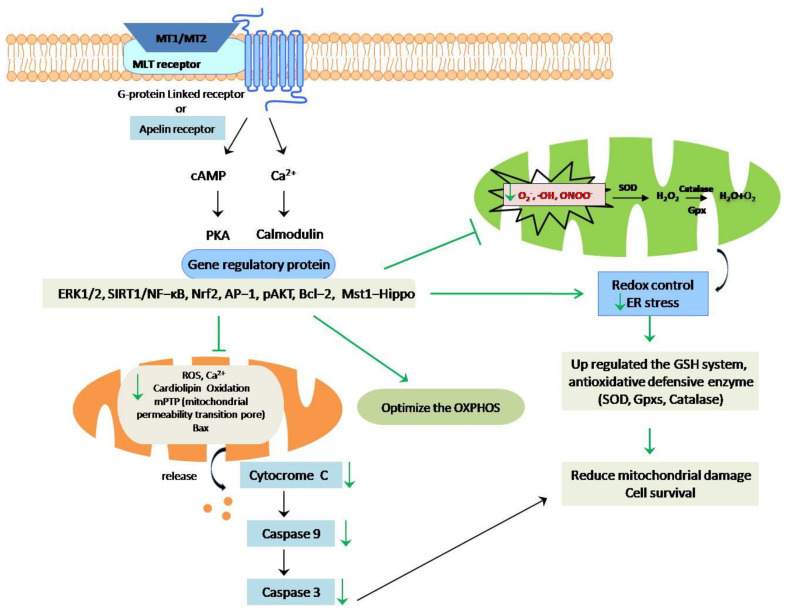
Possible anti-apoptotic pathway of melatonin in case of SARS-CoV-2 infection.

**Table 1:** Autophagic effects of melatonin.

Autophagy	Action of Melatonin	Condition	Reference
Regulation of autophagic effects	↓ LC3-II/LC3-I ratio, p62/SQSTM1, Beclin1, Atg5, Atg12, and Atg16L1 in liver	Rabbit hemorrhagic disease virus (RHDV)	[152]
Inhibition of autophagy	Attenuates CDK5 (cyclin-dependent kinase 5)	MPTP-induced neurotoxicity	[153]
Inhibition of autophagy	↓Caspase-3/12 and LC3-II/LAMP-2/cathepsin B	Kainic acid-induced neurotoxicity in mouse	[154]
Inhibition ofmTOR-dependent autophagy in cells	Restore the senescence marker protein 30 (SMP30), which plays an important role in cellular Ca^2+^ homeostasis	Chronic kidney failure by *p*-cresol toxin	[155]
Inhibition of autophagy	↓LC3-II/LC3-I ratio, Beclin1 protein, p62 protein	Coxsackievirus B3-induced myocarditis	[137]

## Data Availability

Not applicable.
